# Real-World Effectiveness of SARS-CoV-2 Vaccine Booster in Hemodialysis Patients with COVID-19 Receiving Molnupiravir

**DOI:** 10.3390/v15020543

**Published:** 2023-02-16

**Authors:** Po-Chun Chen, Chiang-Chi Huang, Chung-Ming Fu, Yi-Chin Chang, Po-Jung Wu, Wen-Chin Lee, Chien-Te Lee, Kai-Fan Tsai

**Affiliations:** 1 Department of Internal Medicine, Kaohsiung Chang Gung Memorial Hospital and Chang Gung University College of Medicine, Kaohsiung 83301, Taiwan; 2 Division of Nephrology, Department of Internal Medicine, Kaohsiung Chang Gung Memorial Hospital and Chang Gung University College of Medicine, Kaohsiung 83301, Taiwan; 3 Division of Infectious Diseases, Department of Internal Medicine, Kaohsiung Chang Gung Memorial Hospital and Chang Gung University College of Medicine, Kaohsiung 83301, Taiwan; 4 Division of Nephrology, Department of Internal Medicine, Kaohsiung Municipal Feng-Shan Hospital, Kaohsiung 83062, Taiwan

**Keywords:** coronavirus disease 2019, hemodialysis, molnupiravir, real-word effectiveness, SARS-CoV-2 vaccine booster, severe acute respiratory syndrome coronavirus 2

## Abstract

The severe acute respiratory syndrome coronavirus 2 (SARS-CoV-2) vaccine booster is one of the most essential strategies against coronavirus disease 2019 (COVID-19) in the era of emerging variants. However, the effectiveness of SARS-CoV-2 vaccine boosters has not much been investigated in hemodialysis (HD) patients receiving oral antiviral agents. In this retrospective study involving 258 HD patients with COVID-19 receiving molnupiravir, we stratified the study cohort according to vaccination status and compared the baseline characteristics and risks of 30-day composite events (COVID-19-related acute care visits, hospitalization, or mortality) among groups. Our analysis demonstrated that the SARS-CoV-2 vaccine boosters markedly decreased the risk of composite events in HD patients (hazard ratio (95% confidence interval), 0.163 (0.063–0.423) for three vs. ≤ two doses of vaccination, *p* < 0.001; 0.309 (0.115–0.830) for four vs. ≤ two doses of vaccination, *p* = 0.020). The benefits of vaccine boosters were similar between patients receiving mRNA-based and protein-based boosters and between those with post-booster intervals of ≤ 120 and > 120 days. In conclusion, for HD patients with initially mild or asymptomatic COVID-19 receiving molnupiravir, the benefits of SARS-CoV-2 vaccine boosters are prominent, irrespective of booster vaccine types.

## 1. Introduction

As of December 2022, coronavirus disease 2019 (COVID-19), caused by severe acute respiratory syndrome coronavirus 2 (SARS-CoV-2), has resulted in over 600 million reported cases and over 6 million deaths worldwide [1]. Because of the increased transmissibility and ability to evade host immunity, the emerging variants of SARS-CoV-2 still pose non-negligible threats to human health despite advances in pharmaceutical therapies and global vaccination coverage [2]. Among the variants of concern designated by the World Health Organization (WHO), the Omicron variant has become a globally dominant strain and has led to extensive community spread in Taiwan since 2022, with several highly contagious sublineages such as the BA.1, BA.2, BA.3, BA.4, BA.5, and recent XBB [3,4,5]. Although the severity of the COVID-19 illness caused by the Omicron variant appears to have been reduced compared to that in the pre-Omicron era, probably owing to the attenuated virulence of the strain and the pre-existing immunity derived from previous infection and vaccination, its rapid spread has resulted in a cumulative increase in acute care visits, hospitalizations, and mortality events [6,7]. Additionally, there are still concerns regarding the impact of COVID-19 on vulnerable populations, such as immunocompromised patients and those with multimorbidity, who have increased risks of hospitalization and prolonged illness durations, even in the Omicron era [8,9,10,11]. As one of the populations susceptible to this contagious disease, hemodialysis (HD) patients have a remarkably high risk of COVID-19 infection and disease progression owing to the need for regular dialysis visits, multiple comorbidities, and impaired immune responses to pathogens and vaccinations [12,13,14,15]. Taiwan has the highest prevalence of treated end-stage renal disease (ESRD) worldwide, with 3772 cases per million people in 2020, and HD accounted for approximately 90% of renal replacement therapy in the ESRD population in Taiwan [16,17]. Considering the vulnerability of the HD population to COVID-19 infection and the growing burden of ESRD, adequate management of COVID-19 warrants particular attention in this population [18,19,20].

In the era of emerging variants, extensive vaccination, especially booster vaccination, is one of the most essential strategies against COVID-19 [21,22]. Based on results from clinical trials and real-world studies, booster vaccinations, after a two-dose primary vaccine series, can improve immunity against SARS-CoV-2 variants and reduce the risks of hospitalization and mortality in the overall population [23,24,25,26]. Furthermore, previous studies have indicated that two-dose primary vaccination might not be sufficiently protective against SARS-CoV-2 variants in HD patients, and vaccine boosters could be helpful in sustaining immunity against COVID-19 in this vulnerable population [27,28]. Although an extended (three-dose) primary vaccine series can induce a more robust immune response against COVID-19 in immunocompromised patients, a rapid waning of immunity has been reported in the HD population after this vaccine regimen, which also suggests the roles of additional vaccine boosters in HD patients [29,30]. Apart from vaccination, outpatient pharmaceutical treatment is also a key element in the sustained management of COVID-19, which could improve clinical outcomes and ease the burden on the healthcare system [19]. Among the available therapeutics, molnupiravir is a feasible option for HD patients with non-severe COVID-19, which decreases the risk of hospitalization or mortality and can be prescribed without dose adjustment in patients with severe renal impairment [31,32,33]. In recent real-world studies, molnupiravir was well-tolerated and accelerated clinical improvement in non-hospitalized HD patients with COVID-19, thereby highlighting its therapeutic role in this population [34,35]. Since HD patients are considered particularly susceptible to COVID-19, it is important to integrate booster vaccination and effective medical management in this population. Moreover, because of the notable vaccine hesitancy in the HD population, which could be associated with the skepticism about vaccine effectiveness and necessity during the era of emerging variants and available antiviral treatments, the clinical benefit of booster vaccination has become a topic of concern in HD patients receiving oral antiviral agents [36,37,38]. However, the real-world effectiveness of SARS-CoV-2 vaccine boosters has not much been investigated in HD patients with COVID-19, particularly in those receiving molnupiravir. Additionally, the optimal booster vaccine type and durability of booster vaccination in this population are still controversial.

In this retrospective study, we evaluated the real-world effectiveness of SARS-CoV-2 vaccine boosters in HD outpatients who had the first confirmed episode of COVID-19 and received molnupiravir treatment.

## 2. Materials and Methods

### 2.1. Enrollment of the Study Cohort

Patients living in geographically different areas in Taiwan were recruited from the HD unit of the Kaohsiung Chang Gung Memorial Hospital between May 2022 and October 2022. All patients were identified from the COVID-19 registration list in the HD unit, which has been established since January 2020 to record all confirmed COVID-19 cases in the HD unit as per the hospital’s practice. The inclusion criteria were as follows: (1) adult patients (≥20 years of age) who had undergone maintenance HD for at least one month before enrollment, (2) patients who experienced the first confirmed episode of COVID-19 during the study period, and (3) patients displaying a mild illness or asymptomatic status at COVID-19 diagnosis and receiving molnupiravir treatment within five days after COVID-19 onset. COVID-19 diagnosis was based on a positive SARS-CoV-2 test on a nasopharyngeal or throat swab, using a reverse transcription polymerase chain reaction (RT-PCR) or rapid antigen test in compliance with the guidelines of the Taiwan Centers for Disease Control (CDC) [39]. The severity of COVID-19 was categorized according to the definitions provided in the WHO guidelines [40]: mild, moderate, severe, and critical illnesses corresponding to uncomplicated symptoms without pneumonia, non-oxygenation-requiring pneumonia, oxygenation-requiring pneumonia, and life-threatening conditions (such as acute respiratory distress syndrome (ARDS), thromboembolic events, sepsis, or septic shock), respectively. Patients who had a moderate, severe, or critical illness at COVID-19 diagnosis, those who were hospitalized before COVID-19 diagnosis due to other causes, those who were transferred to other HD units within 30 days after disease onset, and those who refused molnupiravir treatment were excluded from the analysis. The study protocol was approved by the Institutional Review Board and Ethics Committee of Chang Gung Medical Foundation, Taipei, Taiwan (IRB No. 202201346B0 and No. 202202368B0) and adhered to the principles of the Declaration of Helsinki. The requirement for informed consent was waived due to the retrospective design and minimal risk of the study.

### 2.2. Vaccination Status of the Study Population

During the study period, the following SARS-CoV-2 vaccines were granted Emergency Use Authorization (EUA) in Taiwan: the Oxford-AstraZeneca (ChAdOx1-S), Moderna (mRNA-1273), Pfizer-BioNTech (BNT162b2), Novavax (NVX-CoV2373), and Medigen (MVC-COV1901) SARS-CoV-2 vaccines [41]. MVC-COV1901 is a domestically developed protein-based SARS-CoV-2 vaccine that was granted EUA in Taiwan owing to its promising immunogenicity and favorable safety profile [42,43]. All these vaccines could be provided for primary and booster vaccinations, while only the mRNA-based (mRNA-1273 and BNT162b2) and protein-based (NVX-CoV2373 and MVC-COV1901) vaccines were recommended as vaccine boosters in patients receiving two-dose ChAdOx1-S primary series. Both homologous and heterologous vaccinations were feasible. The minimum booster interval was 85 days after the latest vaccine dose for the first and second vaccine boosters [44]. The COVID-19 vaccination status of the study population was collected from the treatment records in the HD unit and confirmed using the National Immunization Information System of Taiwan. The vaccination doses, immunized vaccine types, and date of the latest vaccination were recorded. The vaccination status of the enrolled patients was defined according to the latest dose of the SARS-CoV-2 vaccine immunized more than 14 days prior to COVID-19 onset.

### 2.3. Clinical Presentations, Molnupiravir Administration, and Composite Events

Since April 2022, a standardized protocol has been introduced into hospital practice to guide the clinical management of COVID-19 in non-hospitalized HD patients, which was revised from the guidelines of the WHO and Taiwan CDC [40,45,46]. During the COVID-19 pandemic, SARS-CoV-2 diagnostic tests (RT-PCR or rapid antigen test) were performed in HD patients who had symptoms of COVID-19 or were in close contact with COVID-19 patients. For HD patients with a confirmed COVID-19 diagnosis, an investigation of the clinical conditions was conducted by the healthcare workers in the HD unit, including the date of disease onset, initial presentations, disease severity, and requirement for point-to-point transportation services for HD during quarantine. The date of COVID-19 onset corresponded to the date of symptom onset for symptomatic patients; if patients were initially asymptomatic, the date of disease onset was defined as the date of a positive diagnostic test. Patients with initially mild or asymptomatic COVID-19 underwent maintenance HD in the isolation zone of the outpatient HD unit, and those with higher disease severity were transferred to the inpatient department for further treatment. Once the clinical deterioration of COVID-19 was recognized in HD outpatients during follow-up visits, they were referred to the emergency department for additional management and hospitalization evaluation. Since molnupiravir (Merck Sharp and Dohme, Kenilworth, NJ, USA) is an oral antiviral agent granted EUA in Taiwan and available for patients with advanced renal disease, it was offered as a therapeutic option for HD patients with mild or asymptomatic COVID-19, who had a high risk of disease progression [33,46]. For HD patients who did not have contraindications and provided treatment consent, molnupiravir was prescribed at a standard dosage of 800 mg every 12 h for five days [31]. The date of molnupiravir initiation was within five days after COVID-19 onset, as per the guidelines of the Taiwan CDC [46]. The clinical course data within 30 days after COVID-19 onset were recorded in the HD unit as part of the hospital’s practice. In this study, a composite event was defined as a COVID-19-related acute care visit (i.e., visiting the emergency department for additional treatment, except for the initial visit for COVID-19 diagnosis), hospitalization, or mortality event within 30 days after disease onset. Data on COVID-19 diagnosis, clinical presentation, molnupiravir administration, and composite events were extracted from the treatment records in the HD unit.

### 2.4. Collection of Baseline Demographic and Clinical Profiles

The demographic profiles of the enrolled patients were collected from the electronic medical record system and the HD records of the hospital. These included age, sex, body mass index (BMI), HD vintage, smoking habits, use of immunosuppressants, and comorbidities, such as diabetes, hypertension, dyslipidemia, vascular disease, heart failure, liver cirrhosis, lung disease, malignancy, autoimmune disease, and transplantation history. BMI was calculated according to the records of body height and dry body weight within one week before COVID-19 onset. Smoking habits were recorded for those actively smoking within one year before disease onset. Immunosuppressant use within 30 days before disease onset was also recorded (such as steroids with an equivalent dose of ≥5 mg/day prednisolone, calcineurin inhibitors, and disease-modifying antirheumatic drugs). Data on diabetes, hypertension, dyslipidemia, vascular diseases (cardiovascular, cerebrovascular, carotid artery, and peripheral artery diseases), lung diseases (chronic obstructive pulmonary disease, interstitial lung disease, and asthma with regular medication control), and other comorbidities were extracted from the medical records. Furthermore, we also collected the baseline clinical profiles of the enrolled patients from hospital records, including hematological data, blood biochemical profiles, and indicators of dialysis adequacy within one month prior to COVID-19 onset, such as hemoglobin, leukocyte count, platelet count, blood urea nitrogen, serum creatinine, blood electrolytes, liver enzymes, serum albumin, lipid profiles, iron profiles, intact parathyroid hormone level, and Kt/V. Blood laboratory data were derived from pre-dialysis samples midweek (Wednesday or Thursday) or at the last dialysis session of the week (Friday or Saturday) for patients undergoing HD thrice or twice a week, respectively.

### 2.5. Statistical Analysis

Categorical variables are presented as numbers (*n*) with percentages, and continuous variables are presented as medians with interquartile ranges (IQRs). The initial clinical presentation and incidence of composite events were compared among patients with different vaccination statuses using Fisher’s exact test. To evaluate the effects of vaccine boosters on the risk of composite events in the enrolled patients, we stratified the study cohort into three groups: patients receiving ≤ two, three, and four doses of vaccination. The probabilities of event-free status within 30 days after disease onset were plotted as Kaplan–Meier curves and compared among groups using the log-rank test. For patients receiving three and four vaccine doses, the effects of different booster types (i.e., mRNA-based vs. protein-based vaccines as the latest booster) and post-booster intervals (i.e., interval from latest booster to COVID-19 onset, ≤ 120 days vs. > 120 days) on the risk of composite events were also assessed using Kaplan–Meier curves with the log-rank test. To further elucidate the beneficial effects of vaccine boosters on the risk of composite events, patient characteristics were compared among the groups. Categorical variables were analyzed using Fisher’s exact test and continuous variables were analyzed using the Kruskal–Wallis H-test for univariate analysis. Multivariate Cox regression analysis was performed to assess the effectiveness of SARS-CoV-2 vaccine boosters in reducing the risk of 30-day composite events in the study population, adjusting for age, sex, BMI, HD vintage, diabetes, hypertension, vascular disease, heart failure, interval between COVID-19 onset and molnupiravir initiation, and covariates with a *p*-value < 0.1 in the univariate analyses via enter method. Statistical significance was set at a *p*-value < 0.05. Statistical Product and Service Solutions software (version 22.0; IBM, Armonk, NY, USA) was used for all the analyses.

## 3. Results

### 3.1. Characteristics of the Enrolled Patients

**Figure 1:**
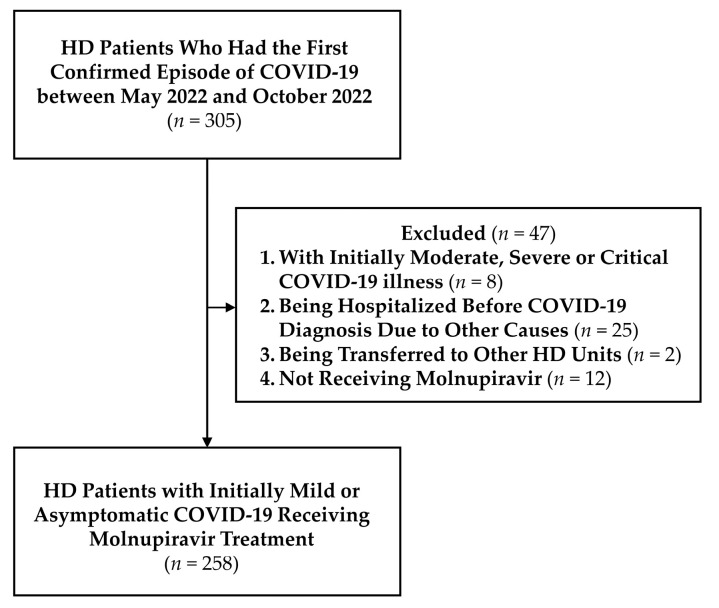
Enrollment of the Study Cohort. COVID-19, coronavirus disease 2019; HD, hemodialysis; *n*, number.

During the study period, 305 patients undergoing maintenance HD in the hospital with the first confirmed episode of COVID-19 were identified. Forty-seven patients were excluded according to the aforementioned exclusion criteria, and 258 patients were enrolled in the analysis (Figure 1). The characteristics of the enrolled patients are summarized in Table 1. The median age of the cohort was 66 years (IQR, 59–71), and 55.81% of the study population were women. The median HD vintage and BMI were 6.25 years (IQR, 2.25–14.17) and 22.98 kg/m^2^ (IQR, 20.51–25.81), respectively. The most common comorbidity was hypertension (91.86%), and 43.80% of the enrolled patients had diabetes. There were 15 (5.81%) patients using immunosuppressants, 10 (3.88%) patients with autoimmune disease, and 9 (3.49%) patients with transplantation history (kidney transplantation, *n* = 8; liver transplantation, *n* = 1). In addition, 28 (10.85%) patients were smokers. Upon COVID-19 diagnosis, all enrolled patients showed a mild illness or asymptomatic status and did not initially require inpatient management. Molnupiravir treatment was administered to all patients within five days of disease onset (median (IQR), 1 (0–1) day).

### 3.2. Presentations and Composite Events among Patients with Different Vaccination Statuses

**Figure 2:**
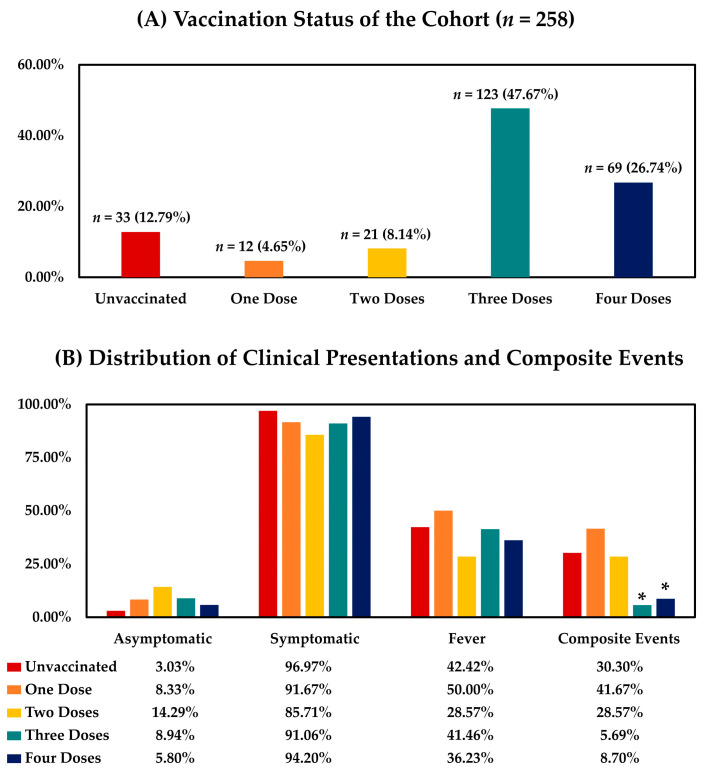
Vaccination Status, Presentations, and Composite Events of the Enrolled HD Patients. (**A**). Vaccination status of the cohort (*n* = 258); (**B**). distribution of clinical presentations and composite events. The composite events were defined as COVID-19-related acute care visits, hospitalizations, or mortality events within 30 days after disease onset. *: *p* < 0.05 compared with unvaccinated patients and those who received one or two doses of vaccination.

In the study population, 123 (47.67%) and 69 (26.74%) patients received three (two-dose primary series and one booster) and four (two-dose primary series and two boosters) doses of SARS-CoV-2 vaccines, respectively, and 21 (8.14%) patients received the two-dose primary vaccine series without a booster before COVID-19 onset. Additionally, 12 (4.65%) patients were immunized with only one vaccine dose, and 33 (12.79%) patients were unvaccinated before disease onset (Figure 2A). The distribution of initial clinical presentations and composite events in the study cohort is shown in Figure 2B. Most enrolled patients (*n* = 238, 92.25%) were symptomatic at diagnosis, including 102 (39.53%) patients describing fever, while 20 (7.75%) patients were initially asymptomatic. The initial presentations were similar among HD patients with different vaccination statuses (unvaccinated vs. one vs. two vs. three vs. four vaccine doses, symptomatic: 96.97% vs. 91.67% vs. 85.71% vs. 91.06% vs. 94.20%, *p* = 0.525; fever: 42.42% vs. 50.00% vs. 28.57% vs. 41.46% vs. 36.23%, *p* = 0.699). During the study period, composite events occurred in 34 enrolled patients, including 4 mortality events. The causes of the composite events included pneumonia (*n* = 8), sepsis (*n* = 6), cardiovascular events such as exacerbation of heart failure and thromboembolic events (*n* = 9), pancreatitis (*n* = 1), and aggravation of COVID-19 symptoms (*n* = 10, such as fever, weakness, anorexia, and drowsiness). The mortality events were ascribed to septic shock (*n* = 1), ARDS (*n* = 1), acute myocardial infarction (*n* = 1), and necrotizing pancreatitis (*n* = 1). There were markedly fewer composite events in HD patients who received at least one vaccine booster compared with those who were not vaccinated or did not receive vaccine booster (unvaccinated vs. one vs. two vs. three vs. four vaccine doses, 30.30% vs. 41.67% vs. 28.57% vs. 5.69% vs. 8.70%, *p* < 0.001).

### 3.3. Risk of Composite Events Stratified by Vaccine Doses, Booster Types, and Post-Booster Intervals in the Enrolled HD Patients

**Figure 3:**
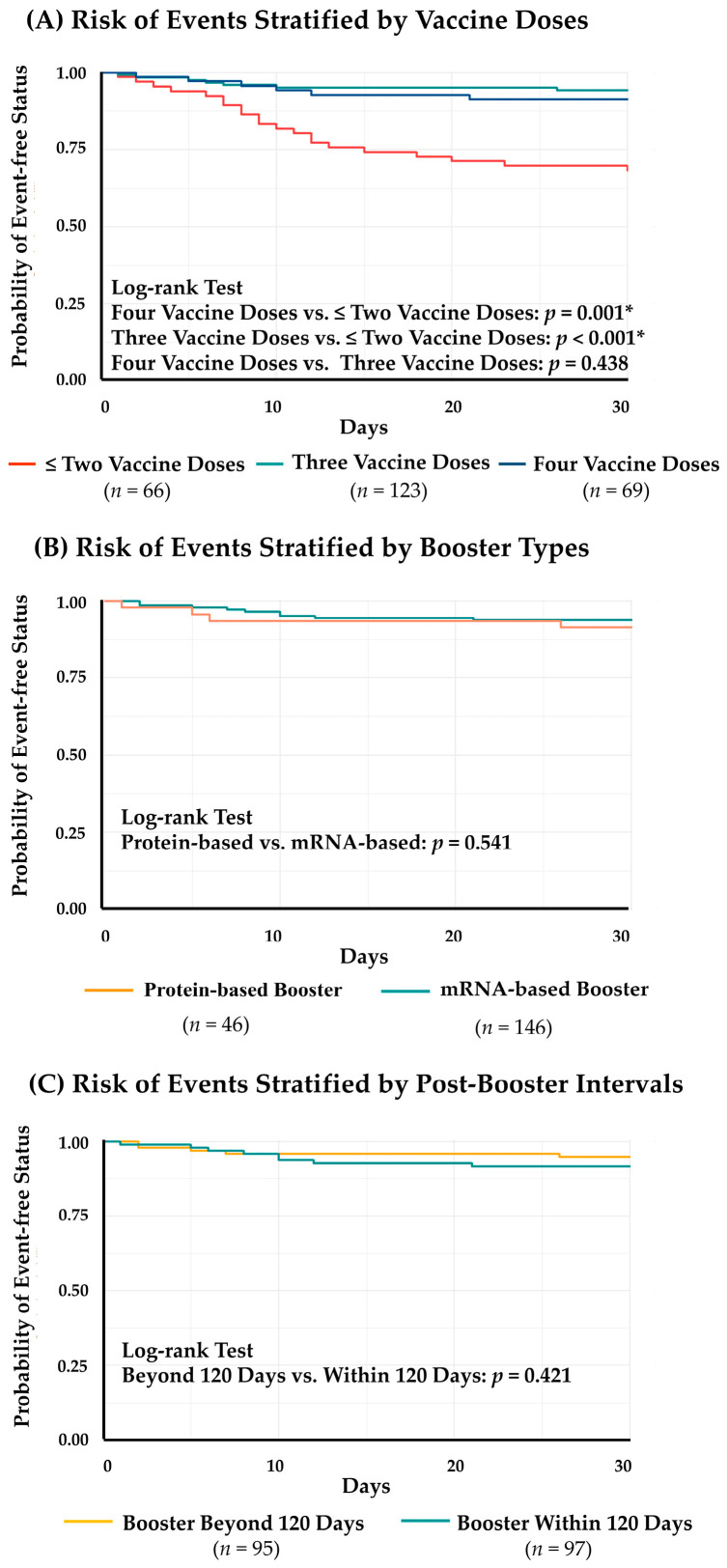
Risk of Composite Events Stratified by Vaccine Doses, Booster Types, and Post-Booster Intervals in the Enrolled HD Patients. (**A**). Risk of events stratified by vaccine doses (*n* = 258); (**B**). Risk of events stratified by booster types (*n* = 192); (**C**). Risk of events stratified by post-booster intervals (*n* = 192). *: *p* < 0.05.

To evaluate the effects of SARS-CoV-2 vaccine boosters on the risk of composite events within 30 days of COVID-19 onset, we stratified the study cohort into three groups: patients who received ≤ two vaccine doses (*n* = 66), three vaccine doses (*n* = 123), and four vaccine doses (*n* = 69). In the group with ≤ two vaccine doses, nine patients visited acute care units without further hospitalization, and 12 patients were hospitalized after acute care visits (including two mortality cases). In the group with three vaccine doses, four patients visited acute care units without hospitalization and three patients required inpatient management after acute care visits (including one mortality case). Furthermore, six patients in the group with four vaccine doses required acute care visits, and four of them were further hospitalized (including one mortality case). The probabilities of event-free status within 30 days of disease onset were plotted as Kaplan–Meier curves and compared using the log-rank test among groups (Figure 3A). Compared to those with ≤ two vaccine doses, patients with three and four doses of vaccination had significantly lower risks of composite events (three vs. ≤ two vaccine doses, *p* < 0.001; four vs. ≤ two vaccine doses, *p* = 0.001). The risks of composite events were similar between patients with three and four doses of vaccination (three vs. four vaccine doses, *p* = 0.483). In the groups that received three and four vaccine doses, 146 patients were immunized with mRNA-based vaccines (mRNA-1273, *n* = 118; BNT162b2, *n* = 28) and 46 were immunized with protein-based vaccines (MVC-COV1901, *n* = 43; NVX-CoV2373, *n* = 3) as the latest booster prior to disease onset. Moreover, the median post-booster interval was 120 days (IQR, 85–139) in the groups that received three and four vaccine doses. In this population, 97 patients received their latest vaccine booster within 120 days before disease onset (median (IQR), 88 (42–109) days), while 95 patients had a post-booster interval beyond 120 days (median (IQR), 140 (129–165) days). Our analysis revealed that the risk of composite events was not significantly altered by booster type and post-booster interval in HD patients who received at least one vaccine booster (mRNA-based vs. protein-based boosters, *p* = 0.541; post-booster intervals ≤ 120 days vs. > 120 days, *p* = 0.421) (Figure 3B,C).

### 3.4. Effects of Vaccine Boosters on Decreasing the Risk of Composite Events

The baseline characteristics of the three groups (i.e., patients who received ≤ two, three, and four doses of SARS-CoV-2 vaccines) are presented in Table 2. The proportions of hypertensive patients (≤ two vs. three vs. four vaccine doses, 98.48% vs. 87.80% vs. 92.75%, *p* = 0.025) and patients with autoimmune disease (≤ two vs. three vs. four vaccine doses, 9.09% vs. 1.63% vs. 2.90%, *p* = 0.037) were lower in the group who received three vaccine doses than in the group who received ≤ two vaccine doses, and history of malignancy was less common in the group with four vaccine doses than in the group with ≤ two vaccine doses (≤ two vs. three vs. four vaccine doses, 31.82% vs. 26.02% vs. 13.04%, *p* = 0.026). Additionally, compared with those who received ≤ two vaccine doses, patients with three and four vaccine doses had higher hemoglobin, serum creatinine, and serum albumin levels (median (IQR), ≤ two vs. three vs. four vaccine doses, 101.00 (91.50–109.25) vs. 106.00 (97.00–114.00) vs. 106.00 (99.50–113.00) g/L, *p* = 0.007; 807.98 (701.45–920.47) vs. 893.72 (771.73–1098.81) vs. 948.53 (820.35–1091.30) μmol/L, *p* < 0.001; 38.50 (35.65–41.05) vs. 40.00 (37.80–42.30) vs. 40.40 (38.45–42.40) g/L, *p* = 0.002, respectively). Furthermore, the alkaline phosphatase level was lower in the group with four vaccine doses (median (IQR), ≤ two vs. three vs. four vaccine doses, 80.00 (62.00–126.00) vs. 75.00 (58.00–103.00) vs. 63.00 (49.00–97.00) U/L, *p* = 0.003), and the HD vintage was slightly longer in the group with three vaccine doses (median (IQR), ≤ two vs. three vs. four vaccine doses, 4.71 (1.29–10.56) vs. 7.67 (2.67–17.25) vs. 5.58 (2.75–10.42) years, *p* = 0.050) than in the group with ≤ two vaccine doses. Other baseline characteristics were similar among the groups.

**Figure 4:**
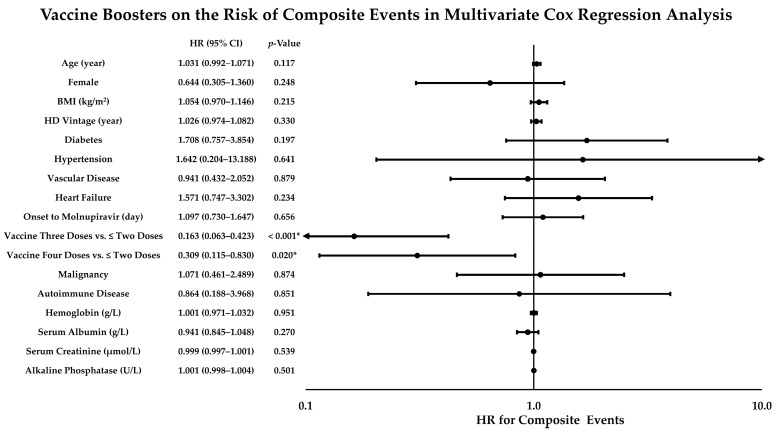
Effects of Vaccine Boosters on Decreasing the Risk of Composite Events in HD Patients with Initially Mild or Asymptomatic COVID-19 Receiving Molnupiravir. Multivariate Cox regression analysis was performed to evaluate the effects of vaccine boosters on the risk of composite events within 30 days of COVID-19 onset, adjusting for age, sex, BMI, HD vintage, diabetes, hypertension, vascular disease, heart failure, interval between COVID-19 onset and molnupiravir initiation, and covariates with a *p*-value < 0.1 in the univariate analyses via the enter method. BMI, body mass index; CI, confidence interval; HR, hazard ratio. *: *p* < 0.05.

To determine the independent predictors of 30-day composite events in HD patients with initially mild or asymptomatic COVID-19 receiving molnupiravir, all covariates with a *p*-value < 0.10 in the univariate analyses were examined in the multivariate Cox regression analysis with the enter method, adjusting for age, sex, BMI, HD vintage, diabetes, hypertension, vascular disease, heart failure, and the interval between COVID-19 onset and molnupiravir initiation (Figure 4). The analysis revealed that SARS-CoV-2 vaccine boosters independently reduced the risk of composite events in the study population, with adjusted hazard ratios of 0.163 (95% confidence interval (CI), 0.063–0.423, *p* < 0.001) for three doses of vaccination and 0.309 (95% CI, 0.115–0.830, *p* = 0.020) for four doses of vaccination compared with ≤ two doses of vaccination.

## 4. Discussion

In this study, we demonstrated that SARS-CoV-2 vaccine boosters were highly protective against COVID-19 in HD patients with an initially mild illness or asymptomatic status and receiving molnupiravir treatment, with an approximately 70–80% reduction in the risk of composite events. The beneficial effects were similar between three and four doses of vaccination, irrespective of the booster type and the post-booster interval, which highlight the durability of booster vaccination in this population. Our findings align with those of previous studies that have assessed the effectiveness of SARS-CoV-2 vaccine boosters in the HD population. In a study conducted by Ducloux et al., a third dose of the BNT162b2 vaccine remarkably enhanced the humoral response against SARS-CoV-2 in COVID-19-naïve HD patients, especially in those with lower responses to a two-dose primary series [47]. Another report in France also revealed that a booster vaccination with the BNT162b2 vaccine could substantially increase anti-SARS-CoV-2 spike protein antibodies in patients undergoing dialysis [48]. Similarly, a prospective study in Thailand indicated that booster vaccination should be considered in HD patients who received an extended primary vaccine series, since the humoral immunity against COVID-19 declined significantly six months after the three-dose primary vaccination in this population [29]. However, most existing studies have focused on the immunogenicity of booster vaccination, and its real-world benefits on the clinical outcomes of COVID-19 have less been investigated in the HD population, particularly in those receiving oral antiviral agents. In addition, although the combination of SARS-CoV-2 vaccines and antiviral treatments is considered beneficial for patients with COVID-19 who are at high risk of disease progression, current reports have yielded mixed results on this issue, and the integrated effects of vaccination and oral antiviral agents have yet to be characterized in HD patients [34,35,49,50]. A previous study utilizing an agent-based mathematical model suggested that combining SARS-CoV-2 vaccines and antiviral treatments could synergistically reduce COVID-19-related hospitalization and mortality in the overall population [49]. Two observational studies in Poland and Japan suggested that combining booster vaccination and molnupiravir treatment was associated with a shorter illness duration and fewer medical requirements in HD patients with COVID-19, although the sample sizes were both small [34,35]. In contrast, another open-label trial addressing the effects of molnupiravir in a highly vaccinated population in the UK (3-dose vaccination, 94%) indicated that hospitalization and mortality might be primarily avoided via extensive vaccination rather than antiviral agents. However, fewer medical consultations and faster clinical recovery were observed in the molnupiravir group, and only 2% of the participants had kidney disease in this trial [50]. Our study demonstrates that SARS-CoV-2 vaccine boosters can further decrease the risk of disease aggravation and medical needs in HD patients with COVID-19 receiving molnupiravir, thereby encouraging extensive booster vaccination in this vulnerable population. Moreover, considering the high prevalence of vaccine hesitancy in the HD population (16.5–34%) in previous studies and the public concerns regarding the effectiveness and necessity of SARS-CoV-2 vaccines during the Omicron era [36,37,38,51], our analysis will serve as an essential reminder of the importance of booster vaccinations, despite the availability of antiviral agents for HD patients.

Although COVID-19 vaccine boosters are highly effective in enhancing immunity against SARS-CoV-2 variants in HD patients, their optimal doses and durability are still uncertain in this population [52]. A previous study in Israel demonstrated that the third dose of the BNT162b2 vaccine induced a more durable humoral response than the second dose in healthcare workers, and the reliable neutralizing capacity for the Omicron variant could persist for at least four months [53]. In a nationwide investigation in the US during the Omicron period, the first vaccine booster was 78% effective in preventing hospitalization even beyond four months after immunization, although the waning of protection was remarkable compared to the initial 91% effectiveness [54]. In contrast, another study reported by Herman-Edelstein et al. indicated that multiple doses of booster vaccination might be required in the HD population owing to their increased risk of breakthrough infection 3–4 months after the first vaccine booster compared to non-dialysis volunteers [55]. Similarly, a report in France recognized an 84.3% decline in anti-SARS-CoV-2 spike protein antibodies six months after the third dose of vaccination in patients undergoing dialysis, although there was no negative conversion of antibody or symptomatic COVID-19 reported during the study period [56]. Despite the current evidence indicating a rapid waning of immunity in the dialysis population, the durability of SARS-CoV-2 vaccine boosters in preventing COVID-19 progression has not much been evaluated in HD patients receiving oral antiviral agents. Our analysis suggested that booster vaccination was similarly beneficial in HD patients receiving three and four doses of vaccination and those with short (≤120 days) and long (> 120 days) post-booster intervals. These observations support the durable effectiveness of SARS-CoV-2 vaccine boosters in HD patients receiving molnupiravir, which highlight their protective roles in this population.

In our investigation, the benefits of SARS-CoV-2 vaccine boosters were similar between HD patients receiving mRNA-based and protein-based vaccine boosters. Although the effectiveness of booster vaccination is well documented in the literature, available evidence is mainly derived from studies assessing mRNA-based vaccines, and the optimal booster vaccine type is yet to be established in the HD population [57]. In a phase 2 trial in the UK, both mRNA-based and protein-based vaccines served as suitable vaccine boosters after homologous ChAdOx1-S or BNT162b2 primary vaccination, but the dialysis population was excluded from the analysis [24]. Another meta-analysis of 38 studies (33 on mRNA-based vaccines and five on viral vector-based vaccines) indicated that the beneficial effects of a third or fourth vaccine dose did not obviously vary with different vaccine types in the HD population, while the effects of protein-based COVID-19 vaccines were not evaluated [57]. Considering their high immunogenicity, favorable safety profiles, and easy deployment, protein-based vaccines are helpful tools to ensure global COVID-19 vaccination coverage [58,59]. Our findings underscore their roles as effective vaccine boosters in the HD population and encourage HD patients to receive booster vaccinations regardless of the available type of vaccine.

Our study has some limitations. As a retrospective study based on the real-world practice of COVID management in HD patients, the study population had an overall high vaccination rate (74.42% received at least one vaccine booster); therefore, we combined patients who received ≤ two vaccine doses as a group for analysis. Additionally, we analyzed the effects of post-booster intervals on clinical outcomes with a cutoff of 120 days owing to the markedly fading immunity beyond four months after booster vaccination in previous studies [54,55]. Further research is required to assess the impact of longer post-booster intervals on vaccine effectiveness in the HD population. Moreover, due to the relatively low COVID-19 prevalence in Taiwan before the Omicron wave since May 2022 and the patient preference for vaccine regimen in real-world settings, the research design focused on patients naïve to COVID-19 before the study period, and the primary vaccinations in the study cohort were all two-dose series, which precluded the assessment of the hybrid immunity and extended (three-dose) primary series [4,29,60]. Although our analysis indicated that the effectiveness of booster vaccination was not altered by the booster type, the sample sizes were unbalanced among different vaccine types because of their variable availability. Moreover, because of the reduced severity of COVID-19 in the Omicron era and the limited number of events [6], the effects of booster vaccination on the individual components of the composite events (i.e., COVID-19-related acute care visits, hospitalization, and mortality) were not evaluated in our study. Finally, next-generation bivalent SARS-CoV-2 vaccines were yet to be widely utilized in HD patients in Taiwan during the study period, and their effectiveness in this population will be a topic of interest for future research [61]. Further large-scale prospective studies are warranted to address these issues and elucidate the optimal strategy for booster vaccination in the HD population. Despite these limitations, our study highlights the real-world effectiveness of SARS-CoV-2 vaccine boosters in HD patients receiving molnupiravir and will be a valuable reference for clinicians and policymakers to encourage extensive booster vaccination in this vulnerable population.

## 5. Conclusions

The benefits of SARS-CoV-2 vaccine boosters are prominent, irrespective of the booster vaccine type, for HD patients with initially mild or asymptomatic COVID-19 receiving molnupiravir. Extensive booster vaccination should be encouraged in the HD population, and further investigation is warranted to enhance our understanding of COVID-19 management in this vulnerable population.

## Figures and Tables

**Table 1:** Characteristics of HD Patients with COVID-19 Receiving Molnupiravir (*n* = 258).

Baseline Demographic Profiles, *n* (%) or median (IQR)	
Age (year)	66 (59–71)
Female	144 (55.81)
BMI (kg/m^2^)	22.98 (20.51–25.81)
HD Vintage (year)	6.25 (2.25–14.17)
Diabetes	113 (43.80)
Hypertension	237 (91.86)
Dyslipidemia	207 (80.23)
Vascular Disease	106 (41.09)
Heart Failure	78 (30.23)
Liver Cirrhosis	23 (8.91)
Lung Disease	27 (10.47)
Smoking	28 (10.85)
Malignancy	62 (24.03)
Autoimmune Disease	10 (3.88)
Immunosuppressant	15 (5.81)
Transplantation History	9 (3.49)
Baseline Clinical Profiles, median (IQR)	
Hemoglobin (g/L)	105.00 (97.00–112.00)
Leukocyte (10^9^/L)	5.80 (4.80–7.13)
Platelet (10^9^/L)	174.50 (141.00–218.25)
Blood Urea Nitrogen (mmol/L)	24.28 (20.71–28.92)
Serum Creatinine (μmol/L)	889.30 (750.96–1051.30)
Kt/V	1.56 (1.37–1.75)
Blood Total Calcium (mmol/L)	2.40 (2.24–2.55)
Blood Phosphorus (mmol/L)	1.65 (1.36–1.94)
Blood Potassium (mmol/L)	4.50 (4.10–4.90)
Blood Bicarbonate (mmol/L)	21.70 (20.08–23.05)
ALT (U/L)	13.00 (9.00–19.00)
Alkaline Phosphatase (U/L)	75.00 (55.75–105.00)
Intact-PTH (ng/L)	204.00 (72.90–427.00)
Transferrin Saturation (%)	29.54 (23.97–37.70)
Serum Albumin (g/L)	39.90 (37.60–41.93)
Total Cholesterol (mmol/L)	3.99 (3.36–4.64)
Onset to Molnupiravir (day)	1 (0–1)

ALT, alanine aminotransferase; BMI, body mass index; COVID-19, coronavirus disease 2019; HD, hemodialysis; intact-PTH, intact parathyroid hormone; IQR, interquartile range; *n*, number.

**Table 2:** Characteristics of HD Patients Who Received ≤ Two, Three, and Four Doses of Vaccination.

	≤Two Vaccine Doses (*n* = 66)	Three Vaccine Doses (*n* = 123)	Four Vaccine Doses (*n* = 69)	*p*-Value
Demographic and Clinical Profiles, *n* (%) or median (IQR)				
Age (year)	66.50 (57.75–72.25)	66.00 (59.00–71.00)	65.00 (58.00–70.00)	0.566
Female	43 (65.15)	68 (55.28)	33 (47.83)	0.129
BMI (kg/m^2^)	21.74 (19.26–25.15)	23.06 (21.01–26.71)	23.44 (20.70–25.40)	0.121
HD Vintage (year)	4.71 (1.29–10.56)	7.67 (2.67–17.25) ^b^	5.58 (2.75–10.42)	0.050 ^#^
Diabetes	28 (42.42)	56 (45.53)	29 (42.03)	0.884
Hypertension	65 (98.48)	108 (87.80) ^a^	64 (92.75)	0.025 *
Dyslipidemia	50 (75.76)	99 (80.49)	58 (84.06)	0.485
Vascular Disease	33 (50.00)	45 (36.59)	28 (40.58)	0.202
Heart Failure	25 (37.88)	33 (26.83)	20 (28.99)	0.272
Liver Cirrhosis	6 (9.09)	11 (8.94)	6 (8.70)	1.000
Lung Disease	7 (10.61)	12 (9.76)	8 (11.59)	0932
Smoking	9 (13.64)	10 (8.13)	9 (13.04)	0.390
Malignancy	21 (31.82)	32 (26.02)	9 (13.04) ^a^	0.026 *
Autoimmune Disease	6 (9.09)	2 (1.63) ^a^	2 (2.90)	0.037 *
Immunosuppressant	7 (10.61)	6 (4.88)	2 (2.90)	0.157
Transplantation History	3 (4.55)	3 (2.44)	3 (4.35)	0.684
Hemoglobin (g/L)	101.00 (91.50–109.25)	106.00 (97.00–114.00) ^a^	106.00 (99.50–113.00) ^a^	0.007 *
Leukocyte (10^9^/L)	5.60 (4.58–7.03)	5.70 (4.90–7.20)	6.00 (4.70–7.30)	0.666
Platelet (10^9^/L)	178.00 (128.50–223.75)	175.00 (141.00–215.00)	173.00 (143.50–215.00)	0.957
Blood Urea Nitrogen (mmol/L)	23.21 (20.35–27.94)	24.99 (20.71–28.92)	24.99 (21.06–29.99)	0.529
Serum Creatinine (μmol/L)	807.98 (701.45–920.47)	893.72 (771.73–1098.81) ^a^	948.53 (820.35–1091.30) ^a^	<0.001 *
Kt/V	1.54 (1.32–1.71)	1.59 (1.41–1.78)	1.54 (1.37–1.72)	0.234
Blood Total Calcium (mmol/L)	2.39 (2.23–2.50)	2.40 (2.23–2.55)	2.43 (2.26–2.58)	0.292
Blood Phosphorus (mmol/L)	1.65 (1.41–2.04)	1.68 (1.32–1.91)	1.58 (1.29–1.91)	0.799
Blood Potassium (mmol/L)	4.60 (4.20–4.93)	4.50 (4.10–4.90)	4.50 (4.10–4.80)	0.423
Blood Bicarbonate (mmol/L)	21.85 (20.18–23.05)	21.40 (19.80–23.00)	21.90 (20.65–23.35)	0.309
ALT (U/L)	13.50 (8.75–18.00)	13.00 (10.00–20.00)	12.00 (9.00–18.00)	0.299
Alkaline Phosphatase (U/L)	80.00 (62.00–126.00)	75.00 (58.00–103.00)	63.00 (49.00–97.00) ^a^	0.003 *
Intact-PTH (ng/L)	220.50 (84.58–629.00)	160.00 (64.40–498.50)	215.00 (89.45–352.25)	0.565
Transferrin Saturation (%)	29.78 (24.01–39.92)	29.44 (23.33–37.70)	29.60 (24.95–36.35)	0.863
Serum Albumin (g/L)	38.50 (35.65–41.05)	40.00 (37.80–42.30) ^a^	40.40 (38.45–42.40) ^a^	0.002 *
Total Cholesterol (mmol/L)	3.74 (3.30–4.46)	4.14 (3.42–4.74)	3.99 (3.46–4.64)	0.312
Onset to Molnupiravir (day)	1 (0–1)	1 (0–1)	1 (0–1)	0.695

^a^: significantly different compared with patients receiving ≤ two vaccine doses; ^b^: slightly different compared with patients receiving ≤ two vaccine doses. *: *p* < 0.05; ^#^: *p* < 0.10.

## Data Availability

All data generated in this study are available from the corresponding author (b9302095@cgmh.org.tw) upon reasonable request, according to the research regulations of the hospital.
